# Association between Cesarean section and neurodevelopmental disorders in a Japanese birth cohort: the Japan Environment and Children’s Study

**DOI:** 10.1186/s12887-023-04128-5

**Published:** 2023-06-19

**Authors:** Taketoshi Yoshida, Kenta Matsumura, Takehiro Hatakeyama, Hidekuni Inadera, The Japan Environment, Children’s Study Group, Michihiro Kamijima, Shin Yamazaki, Yukihiro Ohya, Reiko Kishi, Nobuo Yaegashi, Koichi Hashimoto, Chisato Mori, Shuichi Ito, Zentaro Yamagata, Hidekuni Inadera, Takeo Nakayama, Tomotaka Sobue, Masayuki Shima, Hiroshige Nakamura, Narufumi Suganuma, Koichi Kusuhara, Takahiko Katoh

**Affiliations:** 1 Division of Neonatology, Maternal and Perinatal Center, Toyama University Hospital, 2630 Sugitani, Toyama City, 930-0194 Japan; 2 Department of Public Health, Faculty of Medicine, University of Toyama, Toyama, Japan; 3 Toyama Regional Center for JECS, University of Toyama, Toyama, Japan; 4 Graduate School of Medical Sciences Department of Occupational and Environmental Health, Nagoya City University, 1 Kawasumi, Mizuho-Cho, Mizuho-Ku, Nagoya, Aichi 467-8601 Japan

**Keywords:** Mode of delivery, Autism spectrum disorder, Motor delay, Intellectual disability, Sex difference

## Abstract

**Background:**

The long-term effects of a Cesarean section (CS) birth on child neurodevelopment are of increasing interest. In this study, we examined the associations between mode of delivery and presence of neurodevelopmental disorders in toddlers. Moreover, given that the prevalence of several neurodevelopmental disorders such as autism spectrum disorder (ASD) is known to differ by sex, we also investigated these associations separately in male and female toddlers.

**Methods:**

We investigated 65,701 mother–toddler pairs from the Japan Environment and Children’s Study, a nationally representative children’s cohort study. To investigate the associations between mode of delivery (CS or vaginal delivery) and neurodevelopmental disorders (motor delay, intellectual disability, and ASD) in 3-year-old toddlers as a whole and stratified by sex, we used logistic regression models to calculate adjusted odds ratios (aORs) with 95% confidence intervals (CIs).

**Results:**

The morbidity of ASD at age 3 years was higher for children delivered by CS than those delivered vaginally (aOR 1.38, 95% CI 1.04–1.83). However, no such difference was evident in the case of motor delay or intellectual disability (aOR 1.33, 95% CI 0.94–1.89; aOR 1.18, 95% CI 0.94–1.49, respectively). In the analysis by sex, CS was not associated with increased risk of any of the neurodevelopmental disorders in males, but it was associated with increased risks of motor delay (aOR 1.88, 95% CI 1.02–3.47) and ASD (aOR 1.82, 95% CI 1.04–3.16) in females.

**Conclusions:**

This study provides evidence of significant associations between mode of delivery and neurodevelopmental disorders in early childhood. Females may be more sensitive to the effects of CS than males.

**Supplementary Information:**

The online version contains supplementary material available at 10.1186/s12887-023-04128-5.

## Background

Cesarean section (CS) is a lifesaving and important mode of delivery for neonates and mothers. Its use has increased around the world in recent years [1]. In Japan, CS deliveries as a percentage of total births have doubled since the 1980s and recently exceeded 20% of all deliveries [2]. There are many reports regarding the negative effects of CS on several health outcomes such as obesity, allergy, and asthma [3–5]. The mechanisms underlying the link between CS and future health and mental disorders are believed to be early-term birth [6], altered microbiota [3], decreased serum oxytocin level [7], and use of general anesthesia during CS [8].

The long-term effects of a CS birth on child neurodevelopment are of increasing interest [9–13]. The estimated worldwide prevalence of autism spectrum disorder (ASD) is 0.62% [14], and its incidence has increased 20-fold since the 1980s [15]. A recent study in Japan also reported an increasing trend in ASD diagnoses, from 2.23% (2009) to 3.26% (2014) [16]. There are conflicting results regarding the association between ASD and CS. In the UK, no association was found between CS and ASD [9]. However, in a meta-analysis including 13 studies, CS birth was linked to a greater risk of future ASD (odds ratio (OR) 1.23) than vaginal birth [11]. Although ASD is a highly inheritable disorder with male preponderance, relevant environmental factors may be sex-specific [9]; for example, the indications for CS and the anesthesia method used during CS were found to have some effects on ASD morbidity depending on the infants’ sex [8, 17, 18]. Some environmental factors in the perinatal period may also be involved in ASD morbidity. It is interesting to note that several birth cohort studies have reported increased risks of motor delay and intellectual disability associated with CS, but not the risk of ASD [9, 12, 13, 19]. Zhang et al. investigated the association between CS and neurodevelopmental prognosis in a population exceeding 1 million [10], and their findings suggested that CS was associated with a moderately increased risk of neurodevelopmental disorders in children; however, this risk was mostly explained by familial factors. However, the rate of CS was relatively low (12.4% of all births) among the many children studied and the risks associated with CS stratified by sex were not reported.

The inconsistent results reported about the effects of mode of delivery on subsequent neurodevelopmental problems, including motor delay, intellectual disability, and ASD [9, 10, 12, 19] have mainly been reported for Western populations and the effects in Asian populations are not known. Moreover, there have been very few studies of sex effects on neurodevelopmental disorders according to mode of delivery. Accordingly, in this study, we investigated possible associations between mode of delivery and neurodevelopmental disorders at 3 years of age, using data from the Japan Environment and Children’s Study (JECS), a large nationwide birth cohort study. The associations were also analyzed separately by sex to evaluate the sex-specific relationship between CS and neurodevelopmental disorders.

## Methods

### Study population

This study analyzed data obtained from the JECS, an ongoing birth cohort study that commenced in January 2011. JECS aims to measure the effects of environmental factors on children’s health. The detailed methodology has been previously reported [20–22]. The JECS protocol was reviewed and approved by the Ministry of the Environment’s Institutional Review Board on Epidemiological Studies and by the ethics committees of all participating institutions. The JECS was conducted after obtaining written informed consent from all participants.

**Fig. 1:**
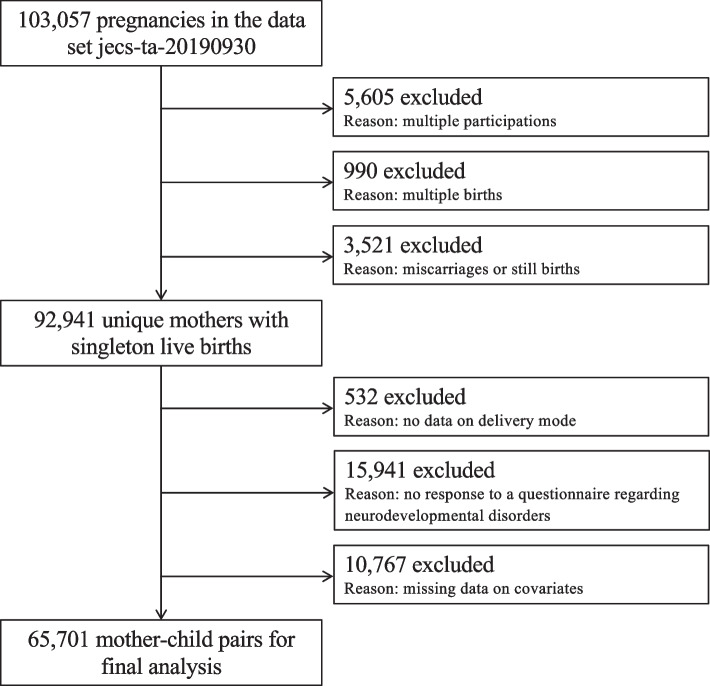
Participant flow diagram

Briefly, pregnant women from 15 regional centers in Japan were recruited over a 3-year period, from 2011 to 2014, and data for 103,057 pregnancies were collected for analysis from the jecs-qa-20210401 (jecs-ta-20190930) datasets, released in April 2021. In the present study, after excluding multiple participations, multiple births, and miscarriages or stillbirths, this left data for 92,941 women with singleton live births. Data for a further 27,240 women were excluded because of missing information about a history of CS or missing answers about neurodevelopmental disorders. Consequently, data for 65,701 mother–toddler pairs were analyzed in this study (Fig. 1).

### Measurements

Data on confounding factors were collected from self-administered JECS questionnaires completed by mothers during follow-up at 1 month postpartum. Medical data were collected from transcribed medical records and included the mode of delivery (transvaginal or CS delivery), gestational age, and birth weight. Data was also collected for mothers’ answers to questions on subsequent JECS questionnaires about whether their child had neurodevelopmental disorders. The neurodevelopmental disorders of motor delay, intellectual disability, and ASD were investigated in the present study, because all healthy children in Japan have a medical examination to detect motor delay, language delay, and intellectual disability at 3 years old. The JECS questions asked whether a physician or health professional had ever told them that their child had motor delay, intellectual disability (including language delay), or ASD (including autism, pervasive developmental disorder, and Asperger’s syndrome).

### Statistical analyses

All statistical analyses were performed using SAS version 9.4 (SAS Institute Inc., Cary, NC, USA) and R (version 4.0.5; R Foundation for Statistical Computing, Vienna, Austria). To investigate the associations between mode of delivery and neurodevelopmental disorders, we used logistic regression models to calculate crude odds ratios (cORs) and adjusted odds ratios (aORs) with 95% confidence intervals (CIs), with vaginal delivery as the reference category. We also examined these associations separately in males and females, because sex differences have been reported in the prevalence of ASD. Two-tailed *p*-values < 0.05 were considered statistically significant.

We selected potential confounders as variables likely to affect or be associated with delivery mode or neurodevelopmental disease. For example, it is known that ASD is highly genetic and has many complications such as anxiety, depression, and hyperactivity [23]. Also, the antenatal and perinatal status of the mother is known to have effects on their infant’s motor development [19]. Therefore, we included a large number of a priori-selected potential confounding variables such as the following: maternal age; pre-pregnancy body mass index; parity; history of depression, anxiety disorder, dysautonomia, or schizophrenia; history of physical disease (i.e., cardiac, cerebrovascular, endocrine, autoimmune, renal, digestive, or gynecological disease or cancer); pregnancy complication (i.e., hypertension, diabetes, or kidney disease); marital status; employment status; highest educational level; annual household income; alcohol intake; negative attitude toward pregnancy (i.e., any answers other than “happy” to the question about the mother’s feeling when learning about her pregnancy); child sex; any major congenital anomaly; gestational age; and birth weight. The covariates were categorized according to usual medical practice or common practice in Japan and/or by referring to our previous studies [24], except for the four continuous variables of maternal age, pre-pregnancy body mass index, gestational age, and birth weight for which the best-fitting spline was used as a smoothing function. To assess model fit and multicollinearity, we calculated R-square values and generalized variance inflation factors, respectively.

## Results

### Descriptive results

From 65,701 singleton births, 12,174 children were born by CS and 53,527 children were born by vaginal delivery. Table 1 shows the maternal and child characteristics grouped by mode of delivery. Mothers who had a CS were older and had higher rates of psychiatric and physical diseases than mothers who had a vaginal delivery. Neonates born by CS were younger and smaller at birth than neonates born by vaginal delivery. Regarding morbidity at 3 years of age, motor delay was reported in 184 toddlers (0.28%), intellectual disability in 466 (0.709%), and ASD in 299 (0.455%). Compared with mothers who were included in the analysis (*n* = 65,701), those who were excluded (*n* = 27,240) tended to have a lower education level, to be a current smoker, to be unmarried, to have a lower household income, and to be younger (Supplementary Table 1).

**Table 1:** Characteristics of mothers and children in relation to mode of delivery

	Cesarean section (*n* = 12,174)		Vaginal delivery (*n* = 53,527)	
Variable	n	(%)	n	(%)
Age during pregnancy, y				
Mean ± SD	32.7	± 4.9	31.1	± 4.8
Pre-pregnancy body mass index, kg/m2				
Mean ± SD	21.9	± 3.8	21.0	± 3.0
Parity				
0	5,342	(43.9)	23,264	(43.5)
1	4,631	(38.0)	19,966	(37.3)
≥ 2	2,201	(18.1)	10,297	(19.2)
History of depression, anxiety disorder, dysautonomia, or schizophrenia				
No	10,346	(85.0)	46,198	(86.3)
Yes	1,828	(15.0)	7,329	(13.7)
History of any physical disease^a^				
No	1,682	(13.8)	9,119	(17.0)
Yes	10,492	(86.2)	44,408	(83.0)
Pregnancy complication				
No	9,722	(79.9)	46,022	(86.0)
Yes	2,452	(20.1)	7,505	(14.0)
Marital status				
Married	11,771	(96.7)	51,639	(96.5)
Single	303	(2.5)	1,554	(2.9)
Divorced or widowed	100	(0.8)	334	(0.6)
Employed during early pregnancy				
No	5,502	(45.2)	24,099	(45.0)
Yes	6,672	(54.8)	29,428	(55.0)
Highest education level, y				
≤ 12	4,156	(34.1)	17,464	(32.6)
12 to < 16	5,336	(43.8)	23,178	(43.3)
≥ 16	2,682	(22.0)	12,885	(24.1)
Annual household income, million JPY				
< 4	4,629	(38.0)	20,674	(38.6)
4 to < 6	4,030	(33.1)	18,032	(33.7)
≥ 6	3,515	(28.9)	14,821	(27.7)
Alcohol intake				
Never	4,072	(33.5)	18,047	(33.7)
Former	7,791	(64.0)	33,994	(63.5)
Current(during pregnancy)	311	(2.6)	1,486	(2.8)
Smoking history				
Never	7,072	(58.1)	32,518	(60.8)
Former	4,624	(38.0)	19,158	(35.8)
Current(during pregnancy)	478	(3.9)	1,851	(3.5)
Negative attitude toward pregnancy				
No	11,376	(93.5)	49,743	(92.9)
Yes	798	(6.6)	3,784	(7.1)
Child sex				
Male	6,214	(51.0)	27,438	(51.3)
Female	5,960	(49.0)	26,089	(48.7)
Any major congenital anomaly				
No	11,751	(96.5)	52,467	(98.0)
Yes	423	(3.5)	1,060	(2.0)
Gestational age, week				
Mean ± SD	38.2	± 2.0	39.5	± 1.3
Birth weight, g				
Mean ± SD	2,875.8	± 500.4	3,062.9	± 376.3

^a^physical disease are heart, cerebrovascular, endocrine system, autoimmune, kidney, digestive system, gynecological, cancer disease etc.

### Logistic regression results

In unadjusted analyses, neonates born by CS had a higher risk of having a neurodevelopmental disorder at 3 years of age than neonates born by vaginal delivery (motor delay: cOR 2.53, 95% CI 1.87–3.41; intellectual disability: cOR 1.75, 95% CI 1.43–2.14; ASD: cOR 1.53, 95% CI 1.18–1.98; Table 2). After adjustment for parental demographics, maternal risks and complications, and neonatal characteristics, CS was significantly associated with ASD (aOR 1.38, 95% CI 1.04–1.83), but the association was no longer significant for motor delay (aOR 1.33, 95% CI 0.94–1.89) or intellectual disability (aOR 1.18, 95% CI 0.94–1.49). When we examined the effects of the mode of delivery on neurodevelopmental disorders according to sex, males born by CS showed no increased risk of motor delay (aOR 1.16, 95% CI 0.75–1.80), intellectual disability (aOR 1.16, 95% CI 0.89–1.52), or ASD (aOR 1.24, 95% CI 0.90–1.73) compared with males born by vaginal delivery. However, females born by CS showed significantly higher risks of motor delay (aOR 1.88, 95% CI 1.02–3.47) and ASD (aOR 1.82, 95% CI 1.04–3.16), but not intellectual disability (aOR 1.35, 95% CI 0.88–2.08), compared with females born by vaginal delivery (Table 2). The R-square values for motor delay, intellectual disability, and ASD in adjusted analyses were 0.121, 0.070, and 0.055, respectively. All generalized variance inflation factors were below 1.68, indicating no multicollinearity among any of the covariates.

**Table 2:** The association between mode of delivery and neurodevelopmental and psychiatric disorders

	Cesarean section (*n* = 12,174)	Vaginal delivery (*n* = 53,527)
Motor delay		
Cases, n	67	117
Prevalence, %	0.55	0.219
Crude odds ratio	**2.53 (1.87, 3.41)**	–––
Adjusted^a^ odds ratio	1.33 (0.94, 1.89)	–––
Intellectual disability (including language delay)		
Cases, n	132	334
Prevalence, %	1.084	0.624
Crude odds ratio	**1.75 (1.43, 2.14)**	–––
Adjusted^a^ odds ratio	1.18 (0.94, 1.49)	–––
Autistic spectrum disorder (e.g., Autism, Pervasive developmental disorder, Asperger syndrome)		
Cases, n	77	222
Prevalence, %	0.632	0.415
Crude odds ratio	**1.53 (1.18, 1.98)**	–––
Adjusted^a^ odds ratio	**1.38 (1.04, 1.83)**	–––

Boldface type indicates significance

"–––" represents reference

^a^Adjusted for maternal age, pre-pregnancy body mass index, parity, history of depression, anxiety disorder, dysautonomia, or schizophrenia, history of physical disease, pregnancy complication, marital status, employed, highest education level, annual household income, alcohol intake, smoking status, negative attitude toward pregnancy, child sex, any major congenital anomaly, gestational age, and birth weight

## Discussion

In this large cohort study of 65,701 mother–infant pairs, we found that children who were born by CS had a higher ASD risk at 3 years of age than those born by vaginal delivery. Moreover, females born by CS had higher risks of motor delay and ASD than females born by vaginal delivery. However, males born by CS showed no significantly higher risk of neurodevelopmental disorders.

After adjustment for potential confounding factors, the risk of ASD was 38% higher in children born by CS than in those born by vaginal delivery in this study. In the literature, there are conflicting reports regarding the association between CS and ASD [7, 10, 11, 17]. Previous studies and a meta-analysis indicated that CS increased the risk of ASD [7, 11, 17], which is line with the findings of the present study. However, the large cohort study by Zhang et al. (2021) in Sweden showed no association between CS and ASD [10]. That study investigated the diagnosis of ASD at an average age of 17 years, whereas we investigated this at 3 years of age. This large difference in the time of diagnosis may be a reason for this conflicting finding. Another reason could be the rate of CS and/or the ratio of emergent CS to elective CS. The rate of CS was 12.4% of all births in Zhang et al.’s study [10], while Japan’s CS rate is higher, at more than 20% [2]. The indication of CS might be different between Sweden and Japan. Although we did not investigate whether CS was emergent or elective in present study, emergent CS occurred in more than half of all CS cases in Zhang et al.’s study [10]. Common indications for emergent CS are related to fetal hypoxia, such as fetal distress, placental abruption, and dystocia, whereas those for elective CS are previous CS, cephalopelvic disproportion, and breech presentation, which have few hypoxic effects on the fetus. General anesthesia during CS was found to increase the risk of ASD compared with local anesthesia [8]. Many cases of emergent CS involve general anesthesia, whereas elective CS tends to involve local anesthesia. Moreover, elective CS tends to be conducted earlier, such as at 37–38 gestational weeks of age, when the neonate’s temperature and blood sugar levels are unstable and brain development has been interrupted [25]. Therefore, the ratio of emergent to elective CS would have some impact on the prognosis of infants.

In terms of neurodevelopmental outcomes, there was no difference between males delivered by CS and males delivered vaginally. However, females delivered by CS had higher morbidities of motor delay and ASD than females delivered vaginally (Table 3). Several studies have examined the sex-specific effects of CS on early childhood neurodevelopmental outcomes. For example, emergent CS was found to be associated with an increased rate of ASD in males, whereas elective CS was associated with an increased rate in females [17]. The risk of ASD in females born by CS was twice as high as that for females born by vaginal delivery [18]. Grace et al. reported that younger maternal age, smoking during early pregnancy, and stress during late pregnancy were associated with motor delay in girls [19]. They suggested that boys and girls were differently affected by antenatal and perinatal risk factors, due to sex-specific developmental pathways. The existence of sex differences in CS birth and subsequent health problems has been reported, with increased incidence of acute lymphoblastic leukemia and hepatoblastoma evident in females [26] and increased incidence of respiratory tract infections evident in males [27]. Females are thought to be more sensitive to the long-term effects of CS than males [17]. Sex differences in CS birth-related health problems have not been generalized, however, and the underlying mechanisms have not been elucidated. The sex-specific propensity may be at least partly due to sex chromosomal gene dosage and sex hormone levels. Experimental studies may be needed to clarify the factors associated with sex-specific incidence of ASD.

**Table 3:** The association between mode of delivery and neurodevelopmental and psychiatric disorders according to child sex

Child sex	Male		Female	
	Cesarean section (*n* = 6,214)	Vaginal delivery (*n* = 27,438)	Cesarean section (*n* = 5,960)	Vaginal delivery (*n* = 26,089)
Motor delay				
Cases, n	39	83	28	34
Prevalence, %	0.628	0.303	0.47	0.13
Crude odds ratio	**2.08 (1.42, 3.05)**	–––	**3.62 (2.19, 5.97)**	–––
Adjusted^a^ odds ratio	1.16 (0.75, 1.80)	–––	**1.88 (1.02, 3.47)**	–––
Intellectual disability (including language delay)				
Cases, n	93	245	39	89
Prevalence, %	1.497	0.893	0.654	0.341
Crude odds ratio	**1.69 (1.33, 2.14)**	–––	**1.92 (1.32, 2.81)**	–––
Adjusted^a^ odds ratio	1.16 (0.89, 1.52)	–––	1.35 (0.88, 2.08)	–––
Autistic spectrum disorder (e.g., Autism, Pervasive developmental disorder, Asperger syndrome)				
Cases, n	54	176	23	46
Prevalence, %	0.869	0.641	0.386	0.176
Crude odds ratio	1.36 (1.00, 1.84)	–––	**2.19 (1.33, 3.62)**	–––
Adjusted^a^ odds ratio	1.24 (0.90, 1.73)	–––	**1.82 (1.04, 3.16)**	–––

Boldface type indicates significance

"–––" represents reference

^a^Adjusted for maternal age, pre-pregnancy body mass index, parity, history of depression, anxiety disorder, dysautonomia, or schizophrenia, history of physical disease, pregnancy complication, marital status, employed, highest education level, annual household income, alcohol intake, smoking status, negative attitude toward pregnancy, any major congenital anomaly, gestational age, and birth weight

In this study, the risk of motor delay was 88% higher in females born by CS than in females born by vaginal delivery (Table 3). Females with ASD tend to have additional complications such as sleep disorder, developmental disorder, and emotional problems [28]. ASD in childhood is often complicated by gross and fine motor delay [29–31]. Because females born by CS were more likely to develop ASD in the present study, this result may contribute to the higher rate of motor delay in females born by CS. Previous studies have reported associations of antenatal and perinatal risk factors, such as maternal pre-eclampsia, CS, and low income, with motor delay in childhood, and these perinatal risk factors may have a lasting effect on fetal neurological systems and postnatal motor development [12, 19]. On the other hand, children born by elective CS also showed motor delay at 9 months of age, although this delay had disappeared at 3 years of age [12]. Moreover, although CS birth was found to be associated with less white matter development in widespread regions of the brain and with less functional connectivity, these effects disappeared with age [32]. These reports indicate that children need to be followed up for longer periods to investigate the relationship between CS birth and neurodevelopmental effects.

The strength of our study is that we used data from a very large sample of mother–child pairs from all over Japan, including both rural and urban locations, so our results are likely to be representative of the Japanese general population. Moreover, we conducted the analysis after adjusting for many potential confounders. Nonetheless, our work had several limitations. First, we used mothers’ answers to JECS questions about whether their child had a neurodevelopmental disorder and it was therefore not clear how motor delay, intellectual disability, and ASD were diagnosed. However, every child in this study had received medical examinations several times, so we consider these diagnoses correct. Second is the timing of ASD diagnosis. Mild ASD cannot be detected at 3 years of age. Although the mean age of diagnosis for children with ASD is 4–6 years, clinical signs are usually present by 3 years of age [23, 33, 34]. In Japan, every healthy child has a medical examination at 3 years old to detect motor delay, language delay, and intellectual disability, and ASD is often diagnosed based on clinical signs such as delayed language. Therefore, we performed this investigation at 3 years. Third, no significant relationship was found between mode of delivery and intellectual disability in this study. However, two articles have reported that children born by CS showed reduced cognitive ability at school age [13, 35]. In our study, children were evaluated at 3 years old, which may be too young to be assessed for cognitive ability. We therefore plan to re-evaluate the participants at school age. Finally, we did not include familial factors, which matters because ASD tends to run in families. Although we did not include familial factors in our analysis, we did include many maternal psychological disorders such as depression, anxiety disorder, dysautonomia, and schizophrenia as cofounders, which may support the reliability of our data.

## Conclusion

This study provides evidence of a significant association between mode of delivery and ASD in early childhood and contributes to the growing body of research attempting to identify the healthcare needs of females with autism and individuals with motor delay. Further research is required to determine the mechanisms underlying this association between CS birth and childhood neurodevelopmental delay and to identify the long-term impact on mental health.

## Supplementary Information

**Additional file 1: Supplementary Table 1.** Difference between included and excluded subjects.

## Data Availability

Data are unsuitable for public deposition due to ethical restrictions and the legal framework of Japan. It is prohibited by the Act on the Protection of Personal Information (Act No. 57 of 30 May 2003, amendment 9 September 2015) to publicly deposit the data containing personal information. Ethical Guidelines for Medical and Health Research Involving Human Subjects enforced by the Japanese Ministry of Education, Culture, Sports, Science and Technology and the Ministry of Health, Labour and Welfare also restrict the open sharing of the epidemiologic data. All inquiries about access to data should be sent to: jecs-en@nies.go.jp. The person responsible for handling inquiries sent to this e-mail address is Dr Shoji F. Nakayama, JECS Programme Office, National Institute for Environmental Studies.

## Abbreviation

CS: Cesarean section
ASD: Autism spectrum disorder
JECS: Japan Environment and Children’s Study
