# Supplementary material for: Association between Cesarean section and neurodevelopmental disorders in a Japanese birth cohort: the Japan Environment and Children’s Study
Source: BMC Pediatr. 2023 Jun 19;23:306. doi: 10.1186/s12887-023-04128-5 (PMC10278360; doi:10.1186/s12887-023-04128-5)
Supplement: Supplementary file 1 — Additional file 1: Supplementary Table 1. Difference between included and excluded subjects. [file 12887_2023_4128_MOESM1_ESM.pdf]

Supplementary Table 1. Difference between included and excluded.

	Included (n = 65,701)		Excluded (n = 27,240)	
Variable	n	(%)	n	(%)
Delibery mode				
Vaginal delivery	53,527	(81.5)	21,406	(80.2)
Cesarean section	12,174	(18.5)	5,302	(19.9)
Age during pregnancy, y				
Mean ± SD	31.4	±4.9	30.0	±5.4
Pre-pregnancy body mass index, kg/m^2				
Mean ± SD	21.2	±3.2	21.4	±3.5
Parity				
0	28,606	(43.5)	9,925	(39.9)
1	24,597	(37.4)	9,310	(37.4)
≥2	12,498	(19.0)	5,630	(22.6)
History of depression, anxiety disorder, dysautonomia, or schizophrenia				
No	56,544	(86.1)	22,018	(84.7)
Yes	9,157	(13.9)	3,993	(15.4)
History of any physical disease				
No	10,801	(16.4)	5,242	(20.2)
Yes	54,900	(83.6)	20,769	(79.9)
Pregnancy complication				
No	55,744	(84.8)	21,412	(84.9)
Yes	9,957	(15.2)	3,800	(15.1)
Marital status				
Married	63,410	(96.5)	23,719	(92.7)
Single	1,857	(2.8)	1,509	(5.9)
Divorced or widowed	434	(0.7)	370	(1.5)
Employed during early pregnancy				
No	29,601	(45.1)	11,951	(48.2)
Yes	36,100	(55.0)	12,823	(51.8)
Highest education level, y				
≤12	21,620	(32.9)	11,208	(44.7)
12 to <16	28,514	(43.4)	9,686	(38.6)
≥16	15,567	(23.7)	4,180	(16.7)
Annual household income, million JPY				
<4	25,303	(38.5)	8,666	(45.6)
4 to <6	22,062	(33.6)	5,900	(31.0)
≥6	18,336	(27.9)	4,441	(23.4)
Alcohol intake				
Never	22,119	(33.7)	8,143	(32.8)
Former	41,785	(63.6)	15,994	(64.4)
Current	1,797	(2.7)	718	(2.9)
Smoking history				

Never	39,590	(60.3)	12,763	(51.4)
Former	23,782	(36.2)	10,269	(41.3)
Current	2,329	(3.5)	1,806	(7.3)
Negative attitude toward pregnancy				
No	61,119	(93.0)	23,329	(91.2)
Yes	4,582	(7.0)	2,244	(8.8)
Child sex				
Male	33,652	(51.2)	14,026	(51.5)
Female	32,049	(48.8)	13,196	(48.5)
Any major congenital anomaly				
No	64,218	(97.7)	26,482	(97.2)
Yes	1,483	(2.3)	758	(2.8)
Gestational age, week				
Mean ± SD	39.3	±1.5	39.2	±1.8
Birth weight, g				
Mean ± SD	3,028.2	±408.7	3,005.1	±445.8
